# A multidisciplinary approach to the detection of and response to West Nile virus in the Netherlands between 2020 and 2023: best practices, challenges and opportunities

**DOI:** 10.2807/1560-7917.ES.2026.31.10.2500276

**Published:** 2026-03-12

**Authors:** Pauline A de Best, Marieta Braks, One Health PACT consortium, National West Nile Virus Response team, working group, Aura Timen, Reina S Sikkema, Marion PG Koopmans, One Health PACT Consortium, Andrea Gröne, Judith M.A. van den Brand, Chiara de Bellegarde de Saint Lary, Emmanuelle Münger, Nnomzie Atama, Louella Kasbergen, Henk van der Jeugd, Kiki Streng, Leo Visser, Louie Krol, Maarten Schrama, Patricia Bruijning-Verhagen, Rody Blom, Constantianus Koenraadt, Wim van der Poel, National West Nile Virus Response team, working group, Arjan Stroo, Adolfo Ibanez-Justicia, Bettie Voordouw, Chantal Reusken, Diederik Brandwagt, Eelco Franz, Hein Sprong, Johan Reimerink, Joke van der Giessen, Ragna Opten, Sabiena Feenstra, Hans Zaaijer, Mariet Feltkamp, Heather Graham, Melle Holwerda, Henk van der Jeugd, Judith M.A. van den Brand, Kees van Maanen, Stijn Raven, Vannessa Visser

**Affiliations:** 1 Viroscience, Erasmus University Medical Center, Rotterdam, the Netherlands; 2 Centre for Infectious Disease Control, National Institute for Public Health and the Environment, Bilthoven, the Netherlands; 3 Members of the One Health PACT Consortium are listed as collaborators; 4 Members of the National West Nile Virus Response team, workgroup are listed as collaborators; 5 Department of Primary and Community Care, RadboudUMC, Nijmegen, the Netherlands; 6 Athena Institute, VU University, Amsterdam, the Netherlands; * This author is also a member of the One Health PACT Consortium.; ** This author is also a member of National West Nile Virus Response team, workgroup.

**Keywords:** West Nile virus, One Health, surveillance, response, evaluation

## Abstract

**BACKGROUND:**

In the Netherlands, locally acquired animal and human cases of West Nile virus (WNV) were first identified in 2020 via multidisciplinary WNV monitoring and research activities focusing on mosquitoes, birds, horses, and humans.

**AIM:**

We investigated how different activities contributed to WNV detection and response in the Netherlands between 2020 and 2023, to determine best practices, challenges, and opportunities for improvement.

**METHODS:**

We identified WNV monitoring and research activities in the Netherlands from 2020 to 2023 and analysed their timeliness to detect and react to WNV circulation. An after-action review (AAR) was conducted with national WNV experts to assess best practices and challenges in the multidisciplinary approach.

**RESULTS:**

In 2020, WNV circulation was discovered in an infected bird through a wild live bird research survey and subsequently through mosquito research and monitoring. Thirty-five days after finding the WNV-PCR-positive bird, the first autochthonous human case was uncovered. Between 2021 and 2023, research projects in animals, including sentinel chickens, detected ongoing local enzootic WNV circulation. The AAR highlighted rapid information sharing and interpretation, enabled by multidisciplinary collaborations, as best practice. However, differing institute priorities could sometimes lead to diverging views on follow-up actions.

**CONCLUSION:**

Research and monitoring activities in mosquitoes and animals, particularly wild birds and sentinel chickens, enabled early detection of WNV circulation. Real-time testing could provide early warning of human cases, enabling timely responses. Therefore, these research and monitoring activities should be maintained. Multidisciplinary collaboration enabled rapid detection and response, and addressing remaining challenges could further strengthen effectiveness.

**Key public health message**

**What did you want to address in this study and why?**

West Nile virus (WNV) can infect mosquitoes, birds and mammals. Its transmission is seasonal, typically between mid-June to mid-November. In Europe, WNV has recently spread to new areas. In the Netherlands, considered non-endemic for WNV, mosquito, bird, and human surveillance enabled the first local WNV identifications in 2020. To improve early warning systems, we assessed how monitoring and research activities contributed to detecting WNV.

**What have we learnt from this study?**

Real-time testing of wild birds provided the earliest signal of local WNV transmission in the Netherlands. Moreover, 35 days after a WNV infected wild bird was found in the country, the first autochthonous human case of WNV was uncovered. Early detection of WNV was possible through multidisciplinary collaboration and integrated surveillance. Rapid information sharing across activities was key to minimise delays and strengthen early warning and response.

**What are the implications of your findings for public health?**

Our findings show that monitoring hosts or vectors of WNV, particularly wild birds, mosquitoes and sentinel chickens can support early identification of WNV circulation. Such early detection may facilitate timely public health responses and allows more time to increase awareness among health professionals. In non-endemic regions, integrated surveillance of these hosts and vectors could strengthen early warning for potential WNV outbreaks.

## Introduction

West Nile virus (WNV) is a zoonotic, mosquito-borne arbovirus from the *Flaviviridae* family [1]. The virus primarily circulates between mosquitoes and birds and can occasionally infect mammals, including humans and horses [2]. *Culex pipiens* is the primary vector species in Europe, whereas birds act as amplifying reservoir hosts. In resident or migratory-bird species that occur in Europe, WNV infections are often asymptomatic, though some species do exhibit symptoms [3]. While humans and horses can develop illness due to WNV, they are considered ‘dead-end’ hosts, as their viraemia levels do not sustain transmission [4]. Although most human infections (80%) are asymptomatic, approximately 20% result in mild disease, such as West Nile fever (WNF) and a small percentage of infected people (1%) may develop a severe illness, known as West Nile neurodegenerative disease (WNND) [2,4]. Among horses with WNV, approximatively 20% exhibit clinical symptoms, and 10% may progress to severe disease [4].

Since the initial report of WNV circulation in Albania in 1958, multiple introductions of different WNV lineages have occurred in Europe. Following these introductions, a growing number of WNV outbreaks affecting birds, equines, and humans across several European regions, and more recently western Europe, indicates not only an increase in case incidence but also a geographical expansion of the virus [5]. As a result, WNV surveillance in currently non-endemic areas is necessary to detect further geographical spread of the virus. Since WNV transmission depends on the presence and activity of competent mosquito vectors, virus circulation follows a seasonal pattern aligned with the mosquito season. In temperate regions such as Europe, WNV transmission typically occurs from mid-June to mid-November, when mosquito activity is highest [6]. Surveillance activities therefore primarily occur in this period to enable timely detection of transmission and potential outbreaks. Furthermore, surveillance systems for WNV often adopt an integrated approach, combining mosquito, wild or domestic bird, horse and human surveillance [7]. Examples of countries employing integrated surveillance are Italy, Spain, Austria, France and the Netherlands [7]. However, deciding when to enhance WNV surveillance in predisposed areas where the virus is considered non-endemic, remains challenging.

In the Netherlands, which is considered non-endemic for WNV, activities to monitor and study WNV transmission are performed by public and animal health institutes with national responsibilities and mandates in surveillance and response for infectious diseases [8]. Additionally, since 2016, One Health research by a consortium of institutes has focused on integrating disease emergence drivers into preparedness and ecological surveys for surveillance and response [9]. These combined efforts led to the first detections of autochthonous WNV infections in birds, mosquitoes and humans in 2020 and evidence for enzootic circulation in the following 2 years. This example of multi-partner surveillance raises the following question: in which way have these separate activities contributed to the detection of WNV and the response to the detection of the virus? Understanding the contribution of each activity can eventually be used to optimise early warning surveillance for WNV. Aside from addressing this question, another objective of this study was to assess best practices and challenges in the collaborative efforts to detect and respond to WNV in the Netherlands.

## Methods

### Document and literature analysis

To assess the activities for the detection of WNV in the Netherlands between 2020 and 2023, a document review was conducted in Google scholar and online libraries of public health institutes. Search terms included ‘Westnijlvirus’, ‘Nederland’, ‘Surveillance’, ‘Rapport’, and ‘Onderzoek’. Additionally, a literature analysis was performed in PubMed. Search terms included ‘West Nile virus’, and ‘Netherlands’. All Dutch and English reports and studies describing WNV detection and response activities within the specified period were included and reviewed in full. The selection process is shown in a Preferred Reporting Items for Systematic Reviews and Meta-Analyses (PRISMA) flowchart in Supplementary material S1. Included studies and reports were categorised in one of three different types of data collection, namely: (i) monitoring activities, as part of (legal) responsibilities in monitoring and response to infectious diseases, including WNV; (ii) longitudinal research surveys, to understand WNV ecology; and (iii) (field)research projects, to assess (risk of) spill-over, spread, transmission routes and ecology of the virus.

### Data compilation

Activities were extracted and summarised per category and host species and the responsible institute(s) performing the activities were identified. We differentiated between national human and animal health (governmental) institutes (G), private institutes (P) with responsibilities in monitoring and response of infectious diseases, and academic institutes whose primary activity is research (R). To assess the contributions of each activity to the detection of, and response to WNV in the Netherlands, data on the number of tested/positive samples were extracted from the identified (grey)literature. Due to the variation in study outcomes and methodologies, a formal quality assessment and meta-analyses were not conducted. Where necessary, additional information was obtained from relevant institutes to supplement the data from reports and scientific literature. Finally, all overviews were reviewed by experts from the field during after action review (AAR, see below), which was conducted through workshops.

### Data analysis

**Figure 1:**
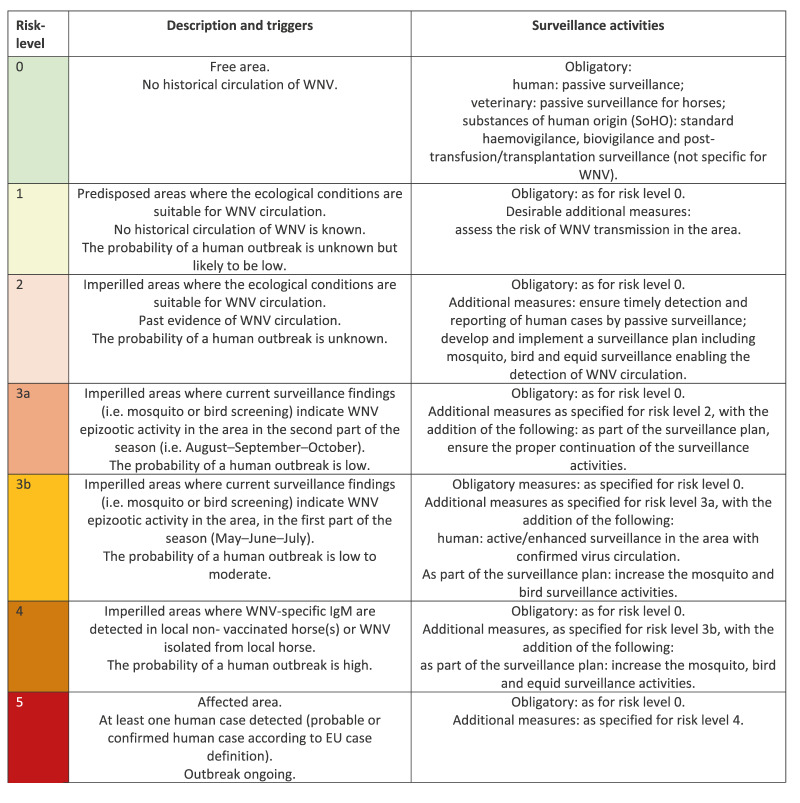
European Centre for Disease Prevention and Control West Nile virus risk-assessment tool (n = 6 risk-levels)

ECDC: European Centre for Disease Prevention and Control; EU: European Union; SoHO: substances of human origin; WNV: West Nile virus.

Timelines showing the contributions of the different activities to key outbreak milestones for WNV detection and response were created based on the compiled data. Time intervals between the execution of key outbreak milestones were assessed and visualised. The definitions of the key outbreak milestones can be found in Supplementary Table S1. Furthermore, using the European Centre for Disease Prevention and Control (ECDC) risk-assessment tool for WNV, the contributions of activities in the three categories to assess the risk-level for WNV in the Netherlands are described [10]. The ECDC tool recognises five risk-levels, which are summarised in Figure 1. Last of all, maps were created showing the locations of all the WNV detections.

Adapted and summarised from the ECDC West Nile virus risk assessment tool [10].

### After action review

Debrief AAR workshops, based on World Health Organization (WHO) guidelines, were conducted on 27 June and 30 July 2024, by stakeholders in the WNV response team [11]. During these self-assessment AAR workshops (from here on referred to as AAR) participants identified best practices and challenges experienced in the period of review (July 2020 through December 2023), for surveillance and outbreak response as defined by the WHO guideline. Participants were selected based on their roles in research, surveillance and outbreak response for WNV in the Netherlands; an overview of participants is presented in Supplementary Table S2. We aimed to include at least one representative per involved institute or department. When members of certain institutes and departments were unable to attend the first workshop, they were invited to join an online shortened AAR.

## Results

Literature and document analysis resulted in the identification of 13 articles [12-24] and three reports [8,25,26]. The Table shows the identified activities and their results per species. A more detailed description of the activities can be found in Supplementary Table S3.

**Table:** Results of multidisciplinary monitoring and response activities, including number of samples tested and number testing positive for West Nile virus infections^a^, the Netherlands, 2020–2023

Group	Activity	Institute	Year, numbers of individuals (or mosquito pools) tested and numbers testing pos.							
			2020		2021		2022		2023	
			Tested	Pos.	Tested	Pos.	Tested	Pos.	Tested	Pos.
**Monitoring activities**										
**Human**	Mandatory notification of WNV infections according to the public health law [13,26]	G	ND	**8^b^**	NA	NA	NA	NA	NA	NA
	Virological testing of patients with suspected WNV infection [13,26]	G and R	93^b^	**3^c^**	399^b^	0^b^	64^b^	0^b^	91^b^	0^b^
	Serological testing of patients with suspected WNV infection [13,26]	G and R	203^b^	**7^c^**	340^b^	0^b^	227^b^	0^b^	285^b^	0^b^
	Syndrome surveillance, CSF surveillance among patients with unexplained neurological complaints with a possible viral cause [13,26]	G and R	101^c^	**4^c^**	160^b^	0^b^	25^b^	0^b^	2^b^	0^b^
	PCR screening of blood donations in the case’s COROP region and the adjacent COROP regions [26]	P	20,596^b^	0^b^	79,641^b^	0^b^	NA	NA	NA	NA
**Horse**	Mandatory notification of WNV infections among equines [26]	G	NA	NA	NA	NA	NA	NA	NA	NA
	Diagnostic testing of horses with suspected WNV based on neurological signs	G and P	3^d^	0^d^	1^b^	0^b^	0^b^	0^b^	4^b^	**1^b,e^**
	Syndrome surveillance of horses with neurological signs [29]	P	0^d^	0^d^	33^b^	0^b^	66^b^	0^b^	63^b^	**1^b,e^**
	Diagnostic testing of horses before export [26]	G and P	68^d^	0^d^	142^b^	0^b^	123^b^	0^b^	127^b^	0^b^
**Mosquito**	Capturing, monitoring (abundance and species) and PCR testing mosquitoes for arboviruses [26	G	1,969^b,f^	**1^b,f^**	1,349^b,f^	0^b^	998^b^	0^b^	708^b^	0^b^
**Longitudinal research surveys**										
**Bird**	Sampling and PCR testing of live birds for arboviruses [22,26]	R	5,640^f^	**7^f^**	9,728^f^	0^f^	12,128^f^	**1^f^**	10,131^g^	0^g^
	Serological testing of live bird serum samples (not real-time tested) [22]	R	728^f^	**18^f^**	813^f^	**8^f^**	1,464^f^	**9^f^**	NA	NA
	Sampling and testing of dead birds for arboviruses (wild and domestic) [22,26]	R and G	405^f^	0^f^	451^f^	0^f^	390^f^	0^f^	208^g^	0^g^
**Mosquito**	Capturing, monitoring (abundance and species) and PCR testing mosquitoes for arboviruses [26]	R	415^b,f^	**5^b,f^**	809^f^	0^f^	466^f^	0^f^	581^g^	0^g^
**(Field) research projects (not real-time tested)**										
**Human**	Seroprevalence study among bird-ringers [18]	R	NA	NA	157	**1^h^**	NA	NA	NA	NA
**Horse**	Seroprevalence study among horses (and dogs) [20]	R	NA	NA	370	**1**	NA	NA	NA	NA
**Dog**	Seroprevalence study among dogs (and horses) [20]	R	NA	NA	258	0	NA	NA	NA	NA
**Wild boars**	Seroprevalence study among wild boars [24]	R	354	**12**	388	**17**	NA	NA	NA	NA
**Bird**	Seroprevalence study among backyard/petting-zoo chickens [21]	R	290	**7**	147	**15**	206	**21**	NA	NA
**Mosquito**	Capturing and testing overwintering mosquitoes for arboviruses [19,23]	R and G	340^i^	0^i^	NA	NA	400^j^	0^j^	NA	NA

COROP: a statistical unit for geographic division of the Netherlands; CSF: cerebrospinal fluid; G: national human and animal health (governmental) institutes with responsibilities in monitoring and response of infectious diseases; NA: not applicable, no samples were collected for the respective activity in this year; ND: not determined; P: private institutes with responsibilities in monitoring of and response to infectious diseases; Pos.: positive; R: academic institutes whose primary objective is research; WNV: West Nile virus.

^a^ Based on PCR and/or serology (protein microarray and confirmation by focus reduction neutralisation test or virus neutralisation test).

^b^ Data from van Ewijk, et al. [26].

^c^ Data from Vlaskamp, et al. [13].

^d^ Information came from a personal communication with Bogt-Kappert (September 2024) at Royal Gezondheidsdienst voor Dieren (Royal GD) or from wider data (not shown) from authors at Wageningen Bioveterinary Research (WBVR). Royal GD and WBVR are the institutes responsible for these activities.

^e^ Same case, initially identified through syndrome surveillance, later confirmed in the diagnostic testing activity.

^f^ Data from Emergence and Dynamics of Usutu and West Nile viruses in the Netherlands 2016–2022, Annex Table S2 Munger et al. [22].

^g^ Information came from wider data (not shown) from authors at Erasmus Medical Centre and from live, dead bird and mosquito monitoring data from bird ringing locations 2023 [22].

^h^ Possible West Nile virus infection, travel-related exposure cannot be excluded.

^i^ Data from Blom et al. [19].

^j^ Data from Koenraadt et al. [23].

### Timeline of events, WNV detections 2020–2023

**Figure 2:**
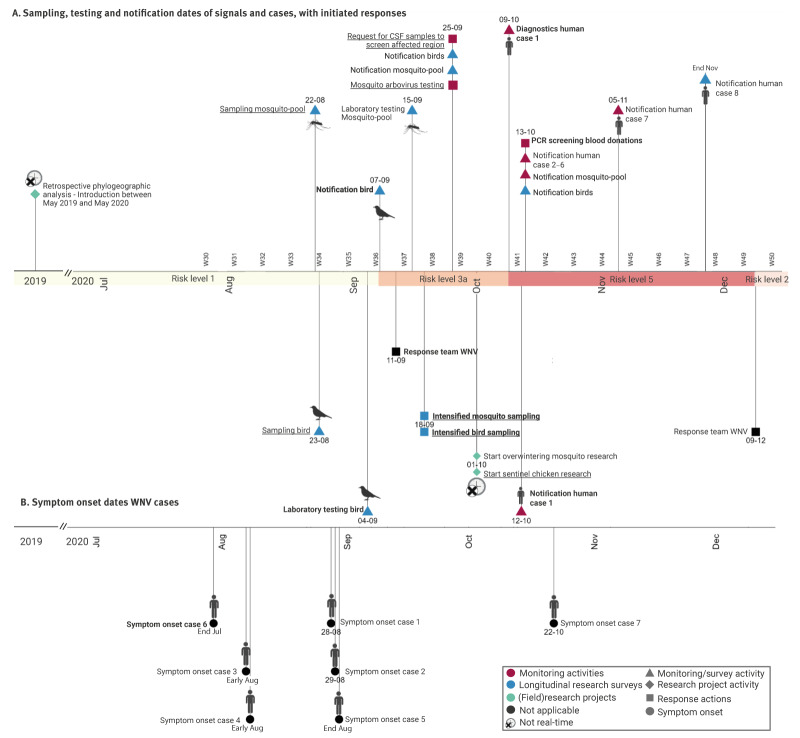
Timeline of events related to West Nile virus detection in the Netherlands in 2020

CSF: cerebrospinal fluid; WNV: West Nile virus.

The first WNV transmission signal, as defined in Supplementary Table S1, was picked up through the live wild bird research survey on 4 September 2020, when a bird testing PCR-positive for WNV was identified [12,22,26] (Figure 2). In response to this detection, research survey efforts were intensified and between September–December 2020, and 1,134 live birds, 52 dead birds, and 54 mosquito-pools were sampled in the area of the first detection of WNV (Table) [13]. The governmental mosquito monitoring was expanded with arbovirus testing, and 809 mosquito-pools were tested in the affected area, of which one was positive for WNV RNA. In October, diagnostic testing of a hospitalised patient with encephalitis confirmed WNND, resulting in the detection of the first autochthonous human case (case definition, Supplementary Table S1) in the Netherlands [13,14,26]. After the first case, seven additional human cases were detected: two WNF cases (case 2 and 4) identified through public health follow-up of contacts of confirmed WNND cases; four WNND cases (case 3,5,6 and 8) through retrospective diagnostic testing of 101 cerebrospinal fluid (CSF) samples from patients with unexplained neurological complaints with a possible viral cause from hospitals in the affected area; and one WNND case (case 7) through diagnostic request [13,15,26] (Table). Overall, all eight cases were symptomatic: six were hospitalised with WNND, and two had WNF. Seven cases lived around the area where the WNV birds and mosquito pools positive for WNV were detected, one case could not be linked to this area [26]. Subsequent PCR screening of 20,596 blood donations from the affected and adjacent COROP regions, did not detect any (asymptomatic) human cases [26]. Overall, seven PCR positive birds, six mosquito-pools, and eight human cases were detected in 2020 [26].

Bold font is used for text relating key milestones; underline font highlights initiatives resulting in WNV detections. The symptom onset date for case 8 was not available, so case 8 is not depicted on panel B; only the date of notification was recorded and is shown in panel A.

**Figure 3:**
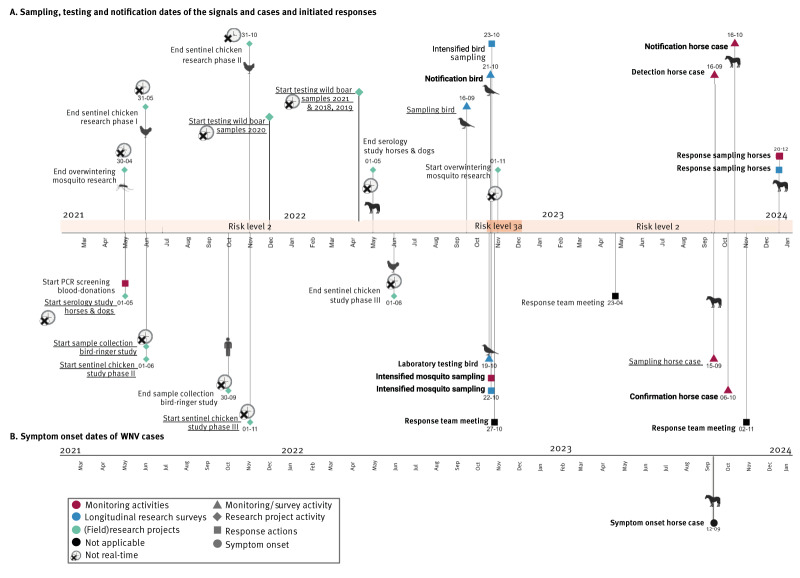
Timeline of research projects, symptom onset, sampling, testing, notification and response dates of the signals and cases, the Netherlands, 2021–2023

In 2021, monitoring activities and longitudinal research surveys in birds, humans, horses and mosquitoes continued, as well as PCR-screening of blood donations, however no signals or cases were detected (Table, Figure 3).

WNV: West Nile virus.

Bold font is used for text relating key milestones; underline font highlights initiatives resulting in WNV detections.

In 2022, PCR-screening of blood donations was discontinued (according to European Union (EU) regulations) [27,28], other activities continued and led to the identification of a WNV-PCR-positive bird through the live wild bird longitudinal research survey (Table). Following the notification of this bird, intensified bird and mosquito sampling was initiated (Figure 3). However, because the notification occurred late in the vector season, there were few mosquitoes, which complicated response sampling [26]. No additional signals or human or animal cases were identified in 2022 (Table).

In 2023, monitoring activities detected WNV infection in a horse, housed in Germany close to the Dutch–German border, treated by a Dutch veterinary practice [29]. Response sampling was conducted on 12 horses that were housed at either the same location as the WNV case or at a premise in the Netherlands where the WNV positive case had participated in a competition during its incubation period. Response testing did not detect any WNV cases in the Netherlands in 2023 (Table, Figure 3).

### Detections and their impact on the ECDC WNV risk-level classification

Before 2020, the WNV ECDC risk-level for the Netherlands was 1 (predisposed areas where the ecological conditions are suitable for WNV circulation). After the detection in birds in 2020, the situation corresponded with risk-level 3a and then 5 after detection of the first human case. When the transmission season ended, the risk-level decreased to level 2 (Figure 1, Figure 2). Retrospective testing detected WNV antibodies in a local bird as early as 2016 and in wild boar in 2018, indicating local circulation before 2020 [22,24]. However, since these samples were tested retrospectively, they did not signal WNV circulation in those years or trigger a risk-level change. Had these samples been tested and notified in real time, the risk-level could have been at level 2 already.

In 2021, no active WNV transmission or cases were detected, and the risk-level remained at level 2. However, after 2021 was over, retrospective serology testing of wild-bird samples collected in 2021 as part of the longitudinal wild bird surveillance, detected WNV antibodies in eight resident birds [22]. Moreover, retrospective testing of samples collected in 2021 in four (field) research projects using serology, in (i) sentinel chickens consisting of backyard/petting-zoo chickens (ii) volunteer bird-ringers (iii) horses, and (iv) wild boars, also detected WNV exposure (Table) [18,20,21,24]. The sentinel chicken study detected WNV exposure in chickens that had tested negative in 2020, strongly suggesting ongoing WNV in the Netherlands in 2021 (Table, Figure 3) [21]. If tested in real-time, this would have altered the risk to level 3b which could have prompted active surveillance for human cases in the area of detection (Figure 1). In the bird-ringer study, serum of one bird ringer showed neutralising antibodies against WNV, however the bird-ringers travel history within and outside of the Netherlands precluded confirmation of the exposure location [18]. The horse study detected WNV neutralising antibodies in a horse without recent travel history or vaccinations, which was thus likely exposed in the Netherlands. However, no IgM was detected so an acute infection was ruled out [20]. Given that neither the horse nor the bird ringer studies had paired samples from before 2021, the exposure could have occurred before 2021, and these detections would not have altered the risk-level in 2021. Finally, WNV antibodies were detected in 17 wild boar samples indicating local WNV circulation, however these samples were not tested in real-time [24].

In 2022, the WNV risk-level increased to 3a after a PCR-confirmed WNV infection was detected in a wild bird and then returned to level 2 after the transmission season ended (Figure 3). Retrospective serology testing of wild bird serum samples collected in 2022 also detected WNV neutralising antibodies in nine birds. 

In 2023, no WNV signals or cases were detected except for the horse case in Germany (close to the Dutch border), circulation of WNV in the Netherlands could not be confirmed, so the risk-level was not affected.

### Geographical distribution of WNV detections

**Figure 4:**
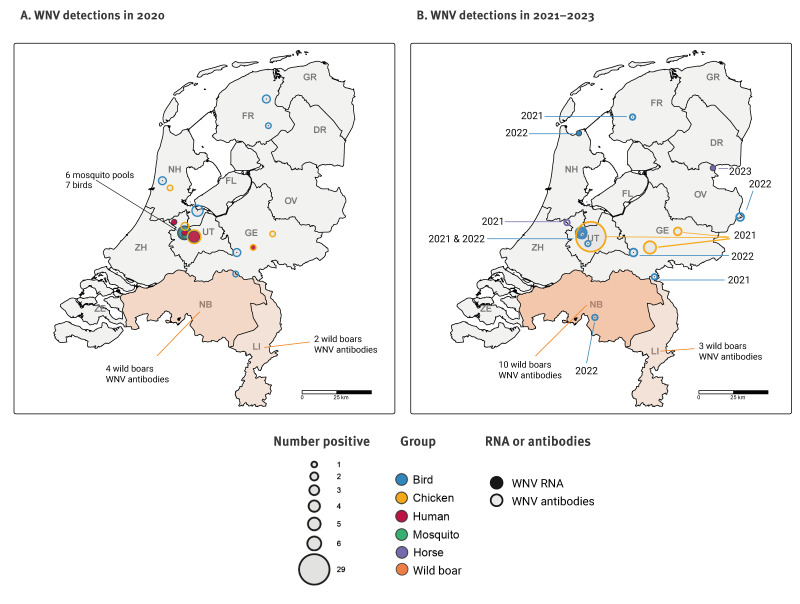
Geographical distribution of the positive West Nile virus (WNV) detections in the Netherlands in (A) 2020 and (B) 2021–2023

WNV: West Nile virus; DR: Drenthe; FL: Flevoland; FR: Friesland; GE: Gelderland; GR: Groningen; LI: Limburg; NB: Noord-Brabant; NH: Noord-Holland; OV: Overijsel; UT: Utrecht; ZH; Zuid-Holland

Real-time monitoring and longitudinal surveillance activities in 2020 detected WNV circulation in the provinces Utrecht and Gelderland (Figure 4a), leading to intensified surveillance efforts around the detection areas. Retrospectively, serological analysis in wild boar and resident wild bird samples also detected WNV antibody signals in other provinces (Figure 4a). Between 2021 and 2023, WNV RNA was detected in a bird in Noord-Holland and a horse in Germany (Figure 4b). Retrospective serological analysis detected WNV in several other provinces throughout the Netherlands in this period, including antibodies in local birds close to the 2023 horse case (Figure 4b).

Circular symbols are coloured per group and sized proportionally to the number of detections. The years represent the sampling year and correspond with Table.

### Time interval analysis, timeliness in detection and response to WNV signal and cases

**Figure 5:**
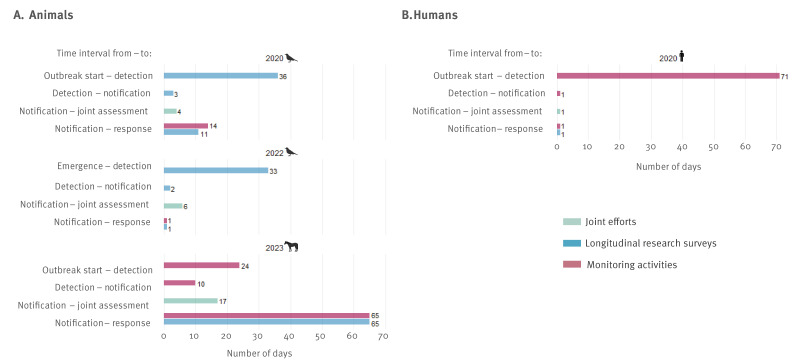
Timeliness of detection and response to West Nile virus (WNV) signals and cases in animals (panel A) and humans (panel B) in 2020, 2022 and 2023

The intervals in Figure 5. are an indication of the timeliness of the detection and response. The longest intervals were observed between the first detection of WNV and the outbreak start, defined as the date of the symptom onset in the primary WNV case (Figure 5, Supplementary Table S1).

WNV: West Nile virus.

Outbreak start/emergence − detection: difference in days between the estimated date of WNV emergence (i.e. date of sampling of the first animal which subsequently tested WNV positive) or outbreak start (date that signs of illness were first observed in a WNV case) and the date of detection; detection − notification: the number of days between first detection and notification of the WNV signal or case to the responsible authority; notification − joint assessment and response: the number of days between notification and joint assessment or other response actions including research.

In 2020, 36 days after the outbreak start (first symptom onset in a human case), a wild bird testing PCR-positive for WNV was discovered (Figure 5b). After the infected bird was found, it took 35 days until routine diagnostics detected the first human case. This detection occurred 71 days after the outbreak start (Figure 5b). Following notification of the bird signal, the first research response action was after 11 days (intensified bird sampling) and the first response by the institutes performing monitoring activities after 14 days (communicating the WNV signal to alert health professionals of potential WNV cases and increase, among others, retrospective CSF diagnostic testing) (Figure 5a). Although the time to detection was longer for the human case, all subsequent milestone intervals were short.

In 2022, the WNV positive bird was sampled in September, but testing was delayed until October, resulting in a time interval of 33 days until detection of the WNV transmission signal. No signals or cases were picked up by monitoring activities in 2022.

In 2023, syndrome surveillance detected a WNV infection in a horse, housed in Germany close to the Dutch–German border. Detection and response actions were initiated by both countries, which complicated confirmation of the infection location and delayed notification of the case. Consequently, this also resulted in longer time intervals for joint assessment and response actions, especially in comparison with 2020 and 2022.

### Best practices and challenges in the multidisciplinary collaboration

AAR participants highlighted that, pre-existing collaborations between institutes within the national zoonoses and response structure, along with collaborative research projects, facilitated information sharing and rapid signal interpretation by experts from various disciplines [9,16]. These pre-existing connections were considered a best practice for response and research, enabling rapid upscaling of response sampling e.g. of mosquitoes.

However, challenges were also identified, including reliance on temporary funding for monitoring and research activities, which could hinder future WNV detection. Furthermore, participants noted that different priorities and responsibilities among government, private, and research institutes led to varying views on for instance public communication and funding allocation. Additionally, caution in (early) sharing of ‘uncertain signals’ (e.g. provisional laboratory signals which might turn out to be false positive or false negative after confirmation testing) was discussed, since it is sometimes unclear which response actions this might (unnecessarily) trigger. Participants suggested developing a shared understanding of surveillance (monitoring and response) priorities, signal certainty and related responses, as well as periodic updates on ongoing research, to enhance trust, encourage more open and rapid signal sharing between institutes, and improve the flow of data and information from research into surveillance, and vice versa.

## Discussion

We assessed the multidisciplinary approach that led to detection of emergence of WNV and subsequent response activities in the Netherlands between 2020 and 2023. We showed that collaborative efforts of national government, private and academic research institutes, detected enzootic WNV circulation and human cases in 2020. The longitudinal live wild bird survey was the timeliest (under condition of real-time testing), leading to the first detection of local WNV circulation, retrospectively determined as 36 days after the outbreak start, (corresponding to when symptom onset first occurred in a human case); comparatively the detection of the first human case occurred 71 days after the outbreak start. The detection of WNV in mosquitoes followed closely the WNV detection in the wild bird (i.e. 11 days after). Early detections in birds and mosquitoes are also described for regions in Italy, where WNV detections in birds and/or mosquitoes tend to occur on average 42 days before the detection of human cases in the same area [30]. A limitation of our timeliness analysis is the reliance on estimated dates of emergence or outbreak onset, as the exact timing is often difficult to determine. As a result, the calculated interval to detection may under- or overestimate the true duration.

Serological testing of wild bird serum samples detected WNV circulation in 2020, 2021 and 2022, and the sentinel chicken study found WNV seroconversions in petting zoo chicken which strongly suggested enzootic circulation of WNV in 2020 and 2021. However, these samples were not tested in real-time, and thus findings were not communicated during the respective transmission seasons. Retrospectively, these results could have been a signal of WNV circulation and might have resulted in intensified surveillance activities or other response (research) actions. Other countries, mainly those with endemic WNV circulation, use sentinel chickens to detect WNV circulation either in studies or as part of the integrated surveillance, including Greece and Italy. In these countries, the use of sentinel chickens led to the detection of WNV circulation even in years with no reported human cases [7,31,32]. Therefore, even with low levels of virus circulation, as seems to be the case in the Netherlands, seroprevalence studies in wild birds and sentinel chickens appeared to be sensitive enough to detect ongoing enzootic circulation [32]. In addition to sentinel chickens, Italy uses poultry pathogen monitoring systems to track WNV serological response, a method that could be explored for sentinel chicken surveillance in the Netherlands [5].

The (field) research studies, using samples from bird-ringers and horses, also detected WNV exposure antibodies. However, paired (previously collected) samples were unavailable to confirm recent, locally acquired infections. Although metadata on travel history and vaccination status were collected, they were insufficient to confirm local exposure. This challenge is commonly described in the literature regarding the use of serology in public health decision-making. Even so, these studies helped to gain a better understanding of WNV circulation in the Netherlands, including potential exposure and asymptomatic infections in horses and humans. Study modifications, such as using repeated measures, could strengthen the integration of findings into public health decision-making by providing clearer signals of local transmission. The seroprevalence study using previously collected samples (collected for other surveillance purposes) from local wild boar populations also detected WNV antibodies through ELISA, a small number of individuals were then confirmed positive by virus neutralisation test, showing a fourfold or greater neutralising titre for WNV compared with Usutu virus and tick-borne encephalitis virus [24]. Since wild boars have a limited home-range these detections indicated local exposure and WNV circulation. While the geographical presence of wild boars in the Netherlands is limited, given that samples are already collected for other purposes their use for future monitoring could be considered and possible implications of signals in wild boars for risk-assessment should be discussed.

Visualisation of the detection locations between 2020 and 2023, showed WNV circulation in several provinces of the Netherlands, some of which were only retrospectively detected in (field) research projects. Since some response actions, including PCR screening of blood donations and retrospective CSF screening, were directed at the affected regions, this is relevant to consider for future detection and response efforts. Additionally, Münger et al. described that less intensive wild bird sampling occurs in some provinces, where WNV circulation consequently might be missed [22]. Increased efforts to sample birds or use other types of samples in these provinces, such as from wild boars, could be considered to improve detection of WNV circulation in areas that are currently less surveyed.

Regardless of the epidemiological situation of WNV in the Netherlands, an integrated approach to detection and response requires collaboration between institutes with different remits. Several best practices and challenges for the collaborative approach to detection and response were identified in the self-assessment AAR. Most importantly, pre-existing collaborations and the official national zoonoses and response structures enabled sharing of data, joint multidisciplinary assessment of signals, and setting-up of response sampling. A study evaluating integrated surveillance approaches in several European countries also identified regular meetings and timely sharing of data among the workgroup members as instrumental to integrated surveillance efforts [7]. It is also argued in the literature that multisectoral collaborations are essential for effective detection and response of emerging infectious diseases [33-35]. The AAR participants indicated that differing priorities and views between academic institutes and institutes with (legal) responsibilities to monitor and respond to infectious diseases, sometimes complicated discussions on which activities should be prioritised based on the available knowledge. This has also been described in the literature as a recurring challenge for integrated collaborative surveillance [36-38]. Several reports suggest strengthening collaborations by clarifying surveillance and response definitions, and addressing lessons learned from previous outbreaks to improve trust and future collaborations, which was also mentioned by the AAR participants [7,37,38]. While the AAR resulted in insightful discussions between participants, a limitation of our study is that the AAR was conducted in two separate sessions due to scheduling constraints among invitees. A single session with all participants might have influenced the dynamics of the discussion and could potentially have resulted in different best practices and challenges.

In terms of the way forward, this study highlights that multidisciplinary collaborations and data sharing enabled early detection and response to WNV circulation in the Netherlands’ provinces. In 2020 human cases were picked up by public health professionals and notified, however with a 71-day delay from the first occurrence of WNV-related illness in a person. Signals of WNV circulation in animals, such as birds and wild boars can serve as an early warning for human and horse cases. Therefore, in addition to the current monitoring activities (mandatory reporting; syndrome surveillance) in humans and horses, we recommend considering structural wild bird and mosquito monitoring and implementation of real-time seroprevalence testing in sentinel chickens, as they could result in early detection of WNV circulation.

## Conclusion

In this study, we evaluated the contribution of different surveillance and response activities to the detection of West Nile virus circulation in the Netherlands between 2020 and 2023. While the number of cases was relatively low, (field) research demonstrated ongoing enzootic WNV circulation from 2020 onwards in several provinces in the Netherlands, indicating that ecological conditions remained suitable for virus circulation despite the absence of human cases after 2020. As the WNV disease impact in the Netherlands may change in the coming years, we recommend regularly evaluating the different activities’ contributions to optimise the surveillance strategy to the local context. This evaluation should include a cost-effectiveness analysis. Furthermore, we recommend follow-up meetings to address and overcome the challenges identified during the AAR to enhance the effectiveness of the surveillance and response systems.

## Data Availability

The data used in this study were obtained from individual studies, which are cited in the manuscript. The datasets generated and analysed during the current study are available from the corresponding author upon reasonable request.

## SupplementaryData

512000 SupplementaryMaterial

## References

[1] Lu L Zhang F Oude Munnink BB Munger E Sikkema RS Pappa S West Nile virus spread in Europe: Phylogeographic pattern analysis and key drivers. PLoS Pathog. 2024;20(1):e1011880. 10.1371/journal.ppat.1011880 38271294 PMC10810478

[2] Colpitts TM Conway MJ Montgomery RR Fikrig E. West Nile Virus: biology, transmission, and human infection. Clin Microbiol Rev. 2012;25(4):635-48. 10.1128/CMR.00045-12 23034323 PMC3485754

[3] Bakonyi T Ferenczi E Erdélyi K Kutasi O Csörgő T Seidel B Explosive spread of a neuroinvasive lineage 2 West Nile virus in Central Europe, 2008/2009. Vet Microbiol. 2013;165(1-2):61-70. 10.1016/j.vetmic.2013.03.005 23570864

[4] Lustig Y Sofer D Bucris ED Mendelson E. Surveillance and diagnosis of west nile virus in the face of flavivirus cross-reactivity. Front Microbiol. 2018;9:2421. 10.3389/fmicb.2018.02421 30369916 PMC6194321

[5] Bakonyi T Haussig JM. West Nile virus keeps on moving up in Europe. Euro Surveill. 2020;25(46):2001938. 10.2807/1560-7917.ES.2020.25.46.2001938 33213684 PMC7678036

[6] European Centre for Disease Prevention and Control (ECDC)European Food Safety Authority (EFSA). Surveillance of West Nile virus infections in humans and animals in Europe, monthly report. EFSA J. 2025;23(7):e9594. 10.2903/j.efsa.2025.9594 40655556 PMC12246792

[7] Gossner CM Marrama L Carson M Allerberger F Calistri P Dilaveris D West Nile virus surveillance in Europe: moving towards an integrated animal-human-vector approach. Euro Surveill. 2017;22(18):30526. 10.2807/1560-7917.ES.2017.22.18.30526 28494844 PMC5434877

[8] Braks MAH, van den Kerkhof JHTC. Westnijlvirus in Nederland, Aanpak surveillance en reponse 2021-2023. Bilthoven: National Institute for Public Health and the Environment; 2021.

[9] One Health PACT. Available from: https://www.onehealthpact.org/.

[10] European Centre for Disease Prevention and Control (EDCD). West Nile virus risk assessment tool. Stockholm: ECDC; 2013. Available from: www.ecdc.europa.eu

[11] World Health Organisation (WHO). Guidance for After Action Review (AAR). Geneva: WHO; 2019.

[12] Sikkema RS Schrama M van den Berg T Morren J Munger E Krol L Detection of West Nile virus in a common whitethroat (*Curruca communis*) and *Culex* mosquitoes in the Netherlands, 2020. Euro Surveill. 2020;25(40):2001704. 10.2807/1560-7917.ES.2020.25.40.2001704 33034280 PMC7545818

[13] Vlaskamp DRM Thijsen SFT Reimerink J Hilkens P Bouvy WH Bantjes SE First autochthonous human West Nile virus infections in the Netherlands, July to August 2020. Euro Surveill. 2020;25(46):1-4. 10.2807/1560-7917.ES.2020.25.46.2001904 33213687 PMC7678035

[14] Taks NJLM Bouvy WH Thijsen SFT Reimerink JHJ Reuskens CBEM Raven CFH. Meningitis op basis van een autochtone infectie met westnijlvirus. [Meningitis due to an autochthonous infection with West Nile virus]. Tijdschr Neurol Neurochir. 2021;122(4):175-9.

[15] Reimerink JHJ Braks MAH Voordouw BCG van den Wijngaard CC van den Kerkhof HCT Reusken CBEM. Westnijlvirus: virologie, epidemiologie, klinische beelden en diagnostiek. [West Nile virus: virology, epidemiology, clinical manifestations, and diagnostics]. Tijdschrift Infectieziekten. 2021;16(4):126-32.

[16] van der Giessen J Vlaanderen F Kortbeek T Swaan C van den Kerkhof H Broens E Signalling and responding to zoonotic threats using a One Health approach: a decade of the Zoonoses Structure in the Netherlands, 2011 to 2021. Euro Surveill. 2022;27(31):2200039. 10.2807/1560-7917.ES.2022.27.31.2200039 35929428 PMC9358405

[17] Braks MAH Duijster JW Stroo CAJ. Het westnijlvirus in 2020 toch in Nederland. [West Nile virus unexpectedly in the Netherlands]. Ned Tijdschr Geneeskd. 2022;166:D6278. 35499516

[18] de Bellegarde de Saint Lary C Kasbergen LMR Bruijning-Verhagen PCJL van der Jeugd H Chandler F Hogema BM Assessing West Nile virus (WNV) and Usutu virus (USUV) exposure in bird ringers in the Netherlands: a high-risk group for WNV and USUV infection? One Health. 2023;16:100533. 10.1016/j.onehlt.2023.100533 37363259 PMC10288042

[19] Blom R Schrama MJJ Spitzen J Weller BFM van der Linden A Sikkema RS Arbovirus persistence in North-Western Europe: Are mosquitoes the only overwintering pathway? One Health. 2022;16:100467. 10.1016/j.onehlt.2022.100467 36531660 PMC9747676

[20] Streng K Hakze-van der Honing RW Graham H van Oort S de Best PA Abourashed A Orthoflavivirus surveillance in the Netherlands: Insights from a serosurvey in horses & dogs and a questionnaire among horse owners. Zoonoses Public Health. 2024;71(8):900-10. 10.1111/zph.13171 39057842

[21] Streng K Atama N Chandler F Blom R van der Jeugd H Schrama M Sentinel chicken surveillance reveals previously undetected circulation of West Nile virus in the Netherlands. Emerg Microbes Infect. 2024;13(1):2406278. 10.1080/22221751.2024.2406278 39295515 PMC11441057

[22] Münger E Atama NC van Irsel J Blom R Krol L van Mastrigt T One Health approach uncovers emergence and dynamics of Usutu and West Nile viruses in the Netherlands. Nat Commun. 2025;16(1):7883. 10.1038/s41467-025-63122-w 40849294 PMC12375057

[23] Koenraadt CJM Münger E Schrama MJJ Spitzen J Altundag S Sikkema RS Overwintering of Usutu virus in mosquitoes, The Netherlands. Parasit Vectors. 2024;17(1):537. 10.1186/s13071-024-06620-y 39716210 PMC11667916

[24] Streng K. Chapter 4. In: From research to preparedness: A study of zoonotic arboviruses in animals, the Netherlands. PhD thesis. Wageningen: Wageningen University; 2024. 10.18174/676149

[25] Braks MAH, Stroo CJ. Westnijlvirus in Nederland: Aanpak Integraal Vectormanagement 2021-2023. [West Nile Virus in the Netherlands: Approach Integrated Vector Management 2021-2023]. Bilthoven: National Institute for Public Health and the Environment; 2021.

[26] van Ewijk C, Feenstra S, ter Bogt-Kappert C, Braks M, Franz E, Geurts van Kessel C, et al. Westnijlvirus in Nederland Surveillance en Respons 2021-2023 Eindrapport. [West Nile Virus in the Netherlands Surveillance and Response 2021-2023 Final Report]. Bilthoven: National Institute for Public Health and the Environment; 2024.

[27] EUR-Lex. Commission Directive 2014/110/EU of 17 December 2014 amending Directive 2004/33/EC as regards temporary deferral criteria for donors of allogeneic blood donations Text with EEA relevance. Official Journal of the European Union. 2014; L366/81. Available from: https://eur-lex.europa.eu/legal-content/EN/TXT/?uri=CELEX%3A32014L0110&qid=1649074114926

[28] Directorate-General for Health and Food Safety. West Nile virus and blood safety introduction to a preparedness plan in Europe. Brussels: European Commission. 6 June 2012. Available from: https://health.ec.europa.eu/document/download/62df551d-a4fa-4a2b-8af5-225506ae9d86_en

[29] Graham H Streng K Henneuse H Fabius L Holwerda M van den Wollenberg L Westnijlvirus dichterbij dan gedacht. [West Nile virus closer than expected]. Tijdschr Diergeneeskd. 2024;149(5):36-9.

[30] Chiari M Prosperi A Faccin F Avisani D Cerioli M Zanoni M West Nile Virus Surveillance in the Lombardy Region, Northern Italy. Transbound Emerg Dis. 2015;62(4):343-9. 10.1111/tbed.12375 25958924

[31] Rizzoli A Rosà R Rosso F Buckley A Gould E. West Nile virus circulation detected in northern Italy in sentinel chickens. Vector Borne Zoonotic Dis. 2007;7(3):411-7. 10.1089/vbz.2006.0626 17767411

[32] Chaintoutis SC Gewehr S Mourelatos S Dovas CI. Serological monitoring of backyard chickens in Central Macedonia-Greece can detect low transmission of West Nile virus in the absence of human neuroinvasive disease cases. Acta Trop. 2016;163:26-31. 10.1016/j.actatropica.2016.07.018 27469618

[33] Koopmans MPG Barton Behravesh C Cunningham AA Adisasmito WB Almuhairi S Bilivogui P One Health High-Level Expert Panel. The panzootic spread of highly pathogenic avian influenza H5N1 sublineage 2.3.4.4b: a critical appraisal of One Health preparedness and prevention. Lancet Infect Dis. 2024;24(12):e774-81. 10.1016/S1473-3099(24)00438-9 39134084 PMC12096394

[34] Dos S Ribeiro C van de Burgwal LHM Regeer BJ. Overcoming challenges for designing and implementing the One Health approach: A systematic review of the literature. One Health. 2019;7:100085. 10.1016/j.onehlt.2019.100085 31016220 PMC6475629

[35] Parkes MW Bienen L Breilh J Hsu LN McDonald M Patz JA All hands on deck: Transdisciplinary approaches to emerging infectious disease. EcoHealth. 2005;2(4):258-72. 10.1007/s10393-005-8387-y

[36] Braks M Giglio G Tomassone L Sprong H Leslie T. Making vector-borne disease surveillance work: New opportunities from the SDG perspectives. Front Vet Sci. 2019;6:232. 10.3389/fvets.2019.00232 31380399 PMC6647909

[37] Archer BN Abdelmalik P Cognat S Grand PE Mott JA Pavlin BI Defining collaborative surveillance to improve decision making for public health emergencies and beyond. Lancet. 2023;401(10391):1831-4. 10.1016/S0140-6736(23)01009-7 37230104 PMC10202415

[38] Hayman DTS Adisasmito WB Almuhairi S Behravesh CB Bilivogui P Bukachi SA One Health High-Level Expert Panel (OHHLEP). Developing One Health surveillance systems. One Health. 2023;17:100617. 10.1016/j.onehlt.2023.100617 38024258 PMC10665171

