# Supplementary material for: A multidisciplinary approach to the detection of and response to West Nile virus in the Netherlands between 2020 and 2023: best practices, challenges and opportunities
Source: Euro Surveill. 2026 Mar 12;31(10):2500276. doi: 10.2807/1560-7917.ES.2026.31.10.2500276 (PMC13077186; doi:10.2807/1560-7917.ES.2026.31.10.2500276)
Supplement: SupplementaryMaterial [file 25-00276_Supplementary_material.pdf]

This supplementary material is hosted by Eurosurveillance as supporting information alongside the article: A multidisciplinary approach to the detection of and response to West Nile virus in the Netherlands between 2020 and 2023: best practices, challenges and opportunities, on behalf of the authors, who remain responsible for the accuracy and appropriateness of the content. The same standards for ethics, copyright, attributions and permissions as for the article apply. Supplements are not edited by Eurosurveillance and the journal is not responsible for the maintenance of any links or email addresses provided therein.

Supplementary material S1

To assess the activities for the detection of West Nile virus (WNV) in the Netherlands between 2020 and 2023, a literature search was conducted in Pubmed, Google scholar and online libraries of public health institutes. We included all Dutch and English-language reports and studies that described WNV detection and response activities relevant to the Netherlands and published between 2020 and December 2024.

In PubMed, we used the following search string: (("West Nile virus"[Mesh]) OR ("West Nile virus"[Title/Abstract])) AND (("Netherlands"[Mesh]) OR ("Netherlands"[Title/Abstract])). Although we did not apply a formal date filter within the PubMed search, we manually screened and included only articles published between 2020 and December 2024, reducing the number of results from 32 to 24.

In Google Scholar, we used the following search terms: "Westnijlvirus" AND "Nederland" AND ("surveillance" OR "rapport" OR "onderzoek"), and limited our inclusion to documents published between 2020 and December 2024. This yielded 34 relevant results.

Additionally, we searched the Rijksinstituut voor Volksgezondheid en Milieu (RIVM) website using the term westnijlvirus. All retrieved results fell within the specified time range.

A PRISMA flowchart illustrating the selection process is included below (figure 1).

Figure 1. *PRISMA flowchart showing identification, screening, eligibility, and inclusion of studies identified in the document and literature analysis.*

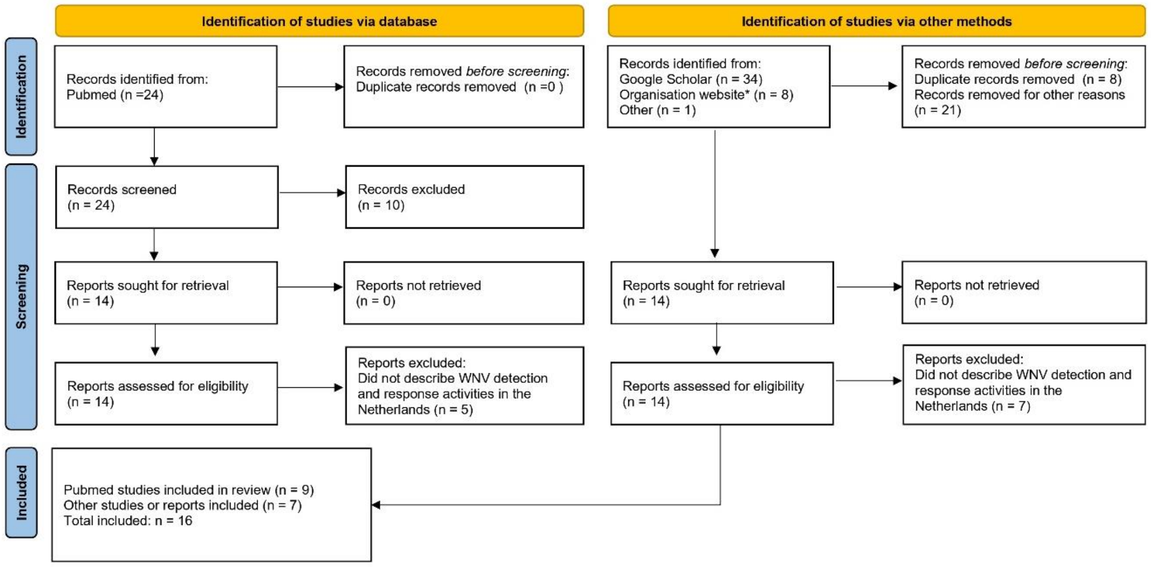

*WNV – West Nile virus; *Rijksinstituut voor Volksgezondheid en Milieu (RIVM) database on their website; PRISMA flowchart is adapted from: Page MJ, et al. BMJ 2021;372:n71. doi: 10.1136/bmj.n71*

Supplementary Table S1

Table 1. *West Nile virus outbreak, key milestone and milestone time interval definitions*

Altered from the World Health organisation key milestone and milestone interval definitions for After Action Reviews, to fit West Nile virus [1].

**WNV definitions**	**Definition**
**Signal of WNV transmission**	A positive mosquito (pool), locally acquired infection in a bird (resident bird or recaptured with previous negative test result), horse (no vaccination history of travel related infection) or human (no travel related infection).
**WNV Case**	a confirmed locally acquired West Nile infection, asymptomatic and symptomatic, in a mammal (human or horse).
**WNV Outbreak**	Signal(s) of widespread WNV circulation (and spread) or increase in spill over in (a region) in the Netherlands; Multiple mosquito-pools or positive birds or a human or horse infection.
**Key milestone**	**Definition**
**Emergence**	Earliest signal of WNV transmission in the Netherlands (mosquito/ bird/ horse or human)
**Outbreak start**	Date of the symptom onset in the primary WNV case or earliest epidemiologically linked case OR date of signal(s) of widespread WNV circulation (spread to other regions)
**First detection**	Date WNV transmission or the outbreak start is first record by any source or in any system
**Notification of detection**	Date WNV transmission signal is first reported by any source to a public health authority responsible for action
**Joint assessment of the notification**	Date the notified signal is jointly assessed by the institutes involved in surveillance and response
**Response actions and research**	Earliest date of any epidemiological or environmental investigations or research in response to the notification, including:<br>• Conduct epidemiologic analysis to determine extent of transmission spread<br>• Performing follow up mosquito trapping and/or increased ecological surveillance<br>• Retrospective testing of encephalitis casus with unknown cause or origin.
**Public communication**	Date of first official release of information to the public from the responsible authority
**Date of outbreak / transmission end**	Date the WNV-transmission season is over, determined by the activity of Culex pipiens and the absence of further cases until the start of the new transmission season
**Milestone interval**	**Definition**

**Interval to detection of emergence or the outbreak start**	Time between the emergence or the outbreak start, and the date on which the first detection of the WNV transmission or outbreak is laboratory confirmed.
**Interval to notification of the signal**	Time between detection of the signal, and notification of the signal to the responsible authority.
**Interval to joint assessment of the notification**	Time between notification of the signal, and joint assessment of the signal by the institutes involved in surveillance and response

Supplementary Table S2

After Action Review participants

***Table 2**. Number of representatives per institute and department in the two After Action Review workshops*

<table>
<tr><th colspan="2">Participants 27th of June 2024</th></tr>
<tr><th>Institute</th><th>Number of representative(s):</th></tr>
<tr><td rowspan="2">Wageningen University and Research (WUR)</td><td>1, Department of entomology</td></tr>
<tr><td>1, Department of Infectious Disease Epidemiology</td></tr>
<tr><td rowspan="4">National Institute for Public Health and the Environment (RIVM)</td><td>2, Department of Diagnostics and laboratory surveillance (IDS)</td></tr>
<tr><td>1, Department of Epidemiology and surveillance infectious diseases (EPI)</td></tr>
<tr><td>1, Department of Communication</td></tr>
<tr><td>1, Department of zoonosis and environmental microbiology (Z&O)</td></tr>
<tr><td>Utrecht University (UU) and
Dutch Wild life Health Centre (DWHC)</td><td>1 representative</td></tr>
<tr><td>Wageningen Bio veterinary Research (WBVR)</td><td>2 representatives</td></tr>
<tr><td>SOVON – bird research</td><td>1 representative</td></tr>
<tr><td>Erasmus Medical Center</td><td>2, Department of Viroscience</td></tr>
<tr><td>Netherlands Food and Consumer Product Safety Authority (NVWA), Centre Monitoring Vectors (CMV)</td><td>1 representative</td></tr>
<tr><td>Leiden University Medical Center</td><td>1, Subdepartment of Infectious Diseases</td></tr>
<tr><th colspan="2">Participants 30th of July 2024</th></tr>
<tr><th>Institute</th><th>Number of representative(s):</th></tr>
<tr><td rowspan="2">National Institute for Public Health and the Environment (RIVM)</td><td>1, Department of zoonosis and environmental microbiology (Z&O)</td></tr>
<tr><td>1, Department National Coordination of Infectious Diseases Control (LCI)</td></tr>
<tr><td>Leiden University</td><td>1, Department Environmental Sciences</td></tr>
<tr><td>Netherlands Food and Consumer Product Safety Authority (NVWA)</td><td>1 representative</td></tr>
<tr><td>Sanquin Blood bank</td><td>2 representatives</td></tr>
<tr><td>Public Health Service Utrecht (GGD)</td><td>1, Department of infectious disease control</td></tr>
<tr><td>Royal GD – Animal health organisation</td><td>1 representative</td></tr>
</table>

Supplementary Table S3

***Table 3**. Monitoring activities, longitudinal research surveys and (field)research projects for WNV, in place between 2020 through 2023 in the Netherlands per species.*

**Species**	**Monitoring activities**	
Human	Mandatory notification of WNV infections according to the public health law disease classification C [2,3]. (Virological and serological testing of patients with suspected WNV infection)	
	Syndrome surveillance, serum or CSF surveillance among patients with unexplained neurological complaints with a possible viral cause [2,3].	
	After the notification of locally acquired WNV cases or if there were WNV cases in the previous year: PCR screening of blood donations in the COROP region of the cases and the adjacent COROP regions [2,3].	
Horse	Mandatory notification of WNV infections among Equines according to the European animal health law. [2,3].	
	Syndrome surveillance of horses with neurological complaints [2,3].	
	Diagnostic testing of horses with suspected WNV based on neurological complaints [2,3].	
	Diagnostic testing of horses before export [3]	
Mosquito	Capturing and monitoring mosquito populations (abundance and species) and after the detection in September 2020, testing of mosquitoes for arboviruses [2,3].	
**Species**	**Longitudinal research surveys**	
Live bird	Sampling of live wild birds by volunteer bird ringers and testing of wild live bird samples for arboviruses [2,3,4].	
Dead bird	Sampling and testing of dead birds for arboviruses (wild and domestic) [2,3].	
Mosquito	Capturing, monitoring (abundance and species) and testing of mosquitoes for arboviruses at bird ringing locations [2,3].	
**Species**	**(field)Research projects**	**Sampling periods**
Human	Seroprevalence study among bird-ringers, a potential high-risk exposure population [2,5].	June 2021 – Sept.2021
Horse	Seroprevalence study among horses (and dogs) [2,3,6].	May 2021 – May 2022
Dog	Seroprevalence study among dogs (and horses) [2,3,6].	May 2021 – May 2022
Wild boar	Seroprevalence study among Wild Boar [7].	Year round from 2018 - 2021
Bird	Sentinel seroprevalence study among backyard and petting-zoo chickens [2,3,8].	Oct. 2020 – May 2021<br>June 2021 – Oct. 2021<br>Nov. 2021 – June 2022
Mosquito	Capturing and testing overwintering mosquitoes for arboviruses [2,3,9].	Oct. 2020 - April 2021<br>& Nov. 2022

CSF: cerebrospinal fluid

COROP: division of the Netherlands into small regions.

References:

1. World Health Organisation (WHO). Guidance for After Action Review (AAR). Geneva; 2019.
2. Braks MAH, van den Kerkhof JHTC. Westnijlvirus in Nederland, Aanpak surveillance en reponse 2021-2023. 2021.
3. C van Ewijk, S Feenstra, C ter Bogt-Kappert, M Braks, E Franz, C Geurts van Kessel, et al. Westnijlvirus in Nederland Surveillance en Respons 2021-2023 Eindrapport. 2024.
4. Münger E, Atama N, van Irsel J, Blom R, Krol L, van den Berg TJ, et al. Emergence and Dynamics of Usutu and West Nile Viruses in the Netherlands, 2016-2022. bioRxiv [Internet]. 2024 Jan 1;2024.12.16.628479.
5. de Bellegarde de Saint Lary C, Kasbergen LMR, Bruijning-Verhagen PCJL, van der Jeugd H, Chandler F, Hogema BM, et al. Assessing West Nile virus (WNV) and Usutu virus (USUV) exposure in bird ringers in the Netherlands: a high-risk group for WNV and USUV infection? One Health. 2023 Jun 1;16
6. Streng K, Hakze-van der Honing RW, Graham H, van Oort S, de Best PA, Abourashed A, et al. Orthoflavivirus surveillance in the Netherlands: Insights from a serosurvey in horses & dogs and a questionnaire among horse owners. Zoonoses Public Health [Internet]. 2024 Jul 26; Available from: https://onlinelibrary.wiley.com/doi/10.1111/zph.13171
7. Streng K, de Freitas Costa E, van Oort S, Hakze van der Honing R, Bron G, Grontvedt CA, et al. Research to preparedness - A study of zoonotic arboviruses in animals, the Netherlands - Chapter 4 Evidence of West Nile virus (WNV) infections in wild boars (Sus scrofa) in the Netherlands, 2018-2021 [Publication in preparation]. 2024.
8. Streng K, Atama N, Chandler F, Blom R, van der Jeugd H, Schrama M, et al. Sentinel chicken surveillance reveals previously undetected circulation of West Nile virus in the Netherlands. Emerg Microbes Infect. 2024 Dec 1;13(1):2406278.
9. Blom R, Schrama MJJ, Spitzen J, Weller BFM, van der Linden A, Sikkema RS, et al. Arbovirus persistence in North-Western Europe: Are mosquitoes the only overwintering pathway? One Health. 2023 Jun 1;16.

Classified as ECDC NORMAL
